# Supplementary material for: Functionalisation of Electrospun Cellulose Acetate Membranes with PEDOT and PPy for Electronic Controlled Drug Release
Source: Nanomaterials (Basel). 2023 Apr 27;13(9):1493. doi: 10.3390/nano13091493 (PMC10180495; doi:10.3390/nano13091493)
Supplement: Supplementary file 1 [file nanomaterials-13-01493-s001.zip › nanomaterials-2307684-supplementary.pdf]

Supplementary information

Functionalisation of Electrospun Cellulose Acetate Membranes with PEDOT and PPy for Electronic Controlled Drug Release

Beatriz Lago [1], Miguel Brito [1], Cristina M. M. Almeida [2,3], Isabel Ferreira [1,*] and Ana Catarina Baptista [1,*]

[1] CENIMAT|I3N, Materials Science Department, School of Science and Technology, NOVA University of Lisbon, 2829-516 Caparica, Portugal
[2] Laboratory of Bromatology and Water Quality, Faculty of Pharmacy, University of Lisbon, 1649-003 Lisboa, Portugal
[3] iMed.UL (Institute for Medicines and Pharmaceutical Sciences, Portugal), Faculty of Pharmacy, University of Lisbon, 1649-003 Lisboa, Portugal
* Correspondence: imf@fct.unl.pt (I.F.); anacbaptista@fct.unl.pt (A.C.B.)

Figure S1 presents the absorbance spectrum (a.u.) of 20 solutions of IBU in SBF with concentrations between 0-20 mg/L in the UV zone (190 nm and 290 nm). The spectra profile is similar for the various concentrations, showing two maximum absorbance peaks at 211 nm and 222 nm.

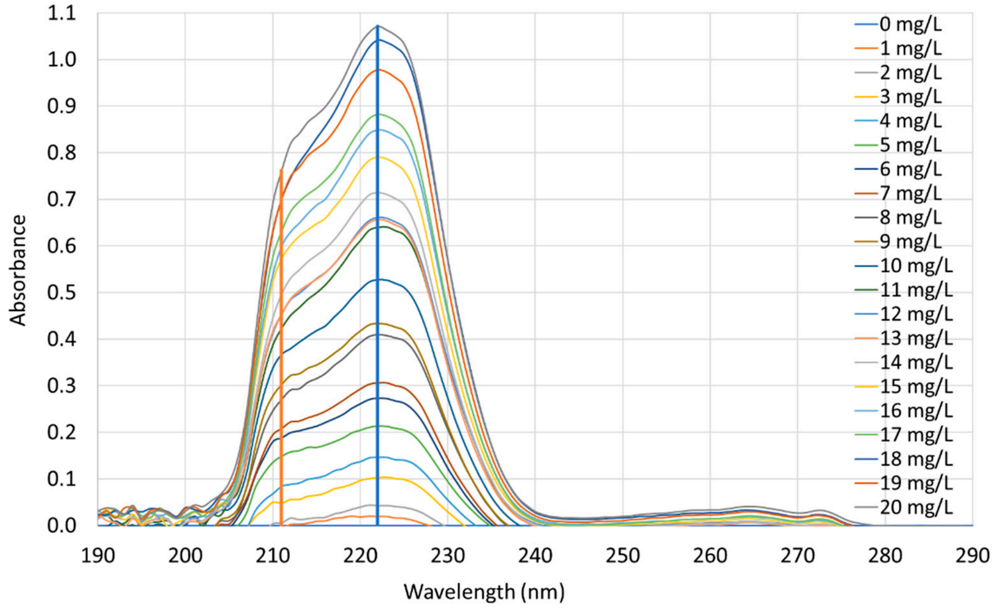

Figure S1. Absorption spectrum for various concentrations of IBU solutions in SBF. Identification of the characteristic peaks.

Figure S2 shows the calibration lines at 211 nm and 222 nm. In the structure of ibuprofen, there are two chromophore groups, the benzene ring and the carboxylic acid, which justify the two absorption maxima in the UV spectrum of Figure S1. During the controlled release tests on the membranes, it was found that there is only an increase in absorption at the wavelength of 211 nm, indicating that one of the chromophore groups has disappeared. Therefore, the IBU molecule reacts with some components of the reaction medium, namely with membrane components that were released into the medium. For this reason, the quantification was performed at 211 nm instead of using the peak at 222 nm, as described by other authors [42].

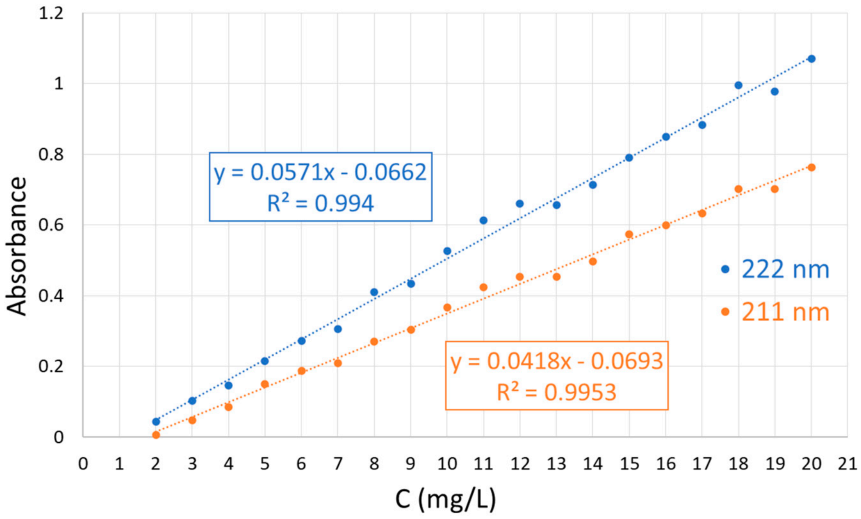

Figure S2. Graph of the calibration curve, estimated for the absorptions recorded at the 211 nm and 222 nm peaks. Equation of the linear regression obtained, with which it is possible to estimate the concentration of drug released into the SBF.

The linear regression of the experimental points obtained shows a good determination coefficient ($R^2 = 0.9953$). In passive and active drug release assays, IBU is quantified through the straight-line equation, absorbance versus concentration obtained for the peak at 211 nm.

Table S1. Optimized MS/MS parameters for chromatographic method.

MS/MS parameters	Acidic Method
Ionization	Electrospray
Q1 and Q3 resolution	0.7
Collision gas	Argon
Sheath, aux and sweep gas	Nitrogen
Sheath gas (Arb)	50
Aux gas (Arb)	12
CID gas (mTorr)	1.5
Vaporizer temperature (°C)	200
Ion transfer tube temperature (°C)	300

Table S2. Mass spectrometer conditions used for IBU analysis by UPLC-MS/MS.

Compound	Ionization mode	t_R window (individual t_R) (min)	MRM transition	Precursor ion – Product ion	Collision energy (V)
IBU	-	1.0 – 4.0 (1.62)	MRM1	205.1 → 161.1	10

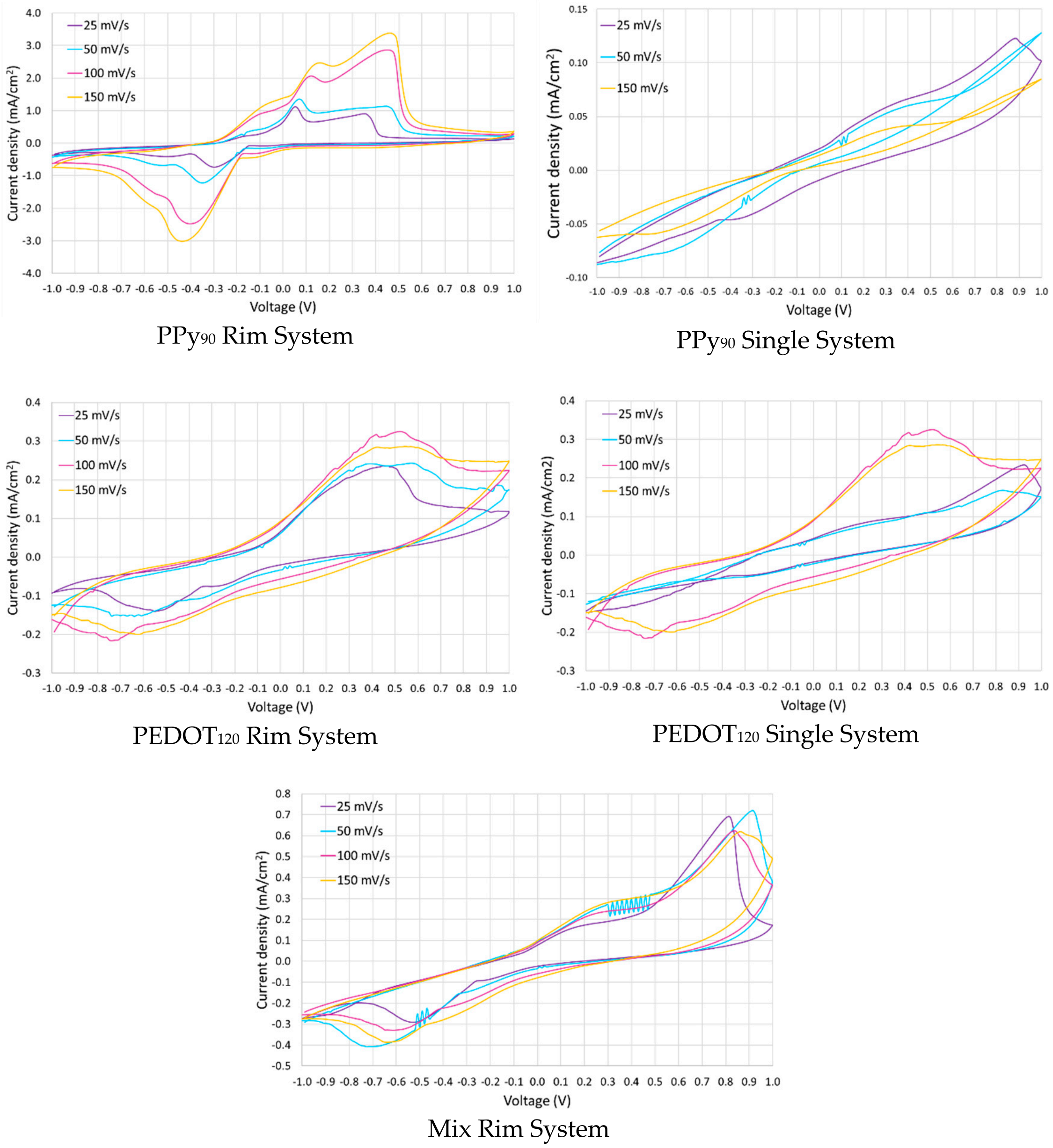

Figure S3. Third cycle of the cyclic voltammogram obtained at different scan rates for the membranes systems studied. All tests were made on the presence of 1mL of SBF.

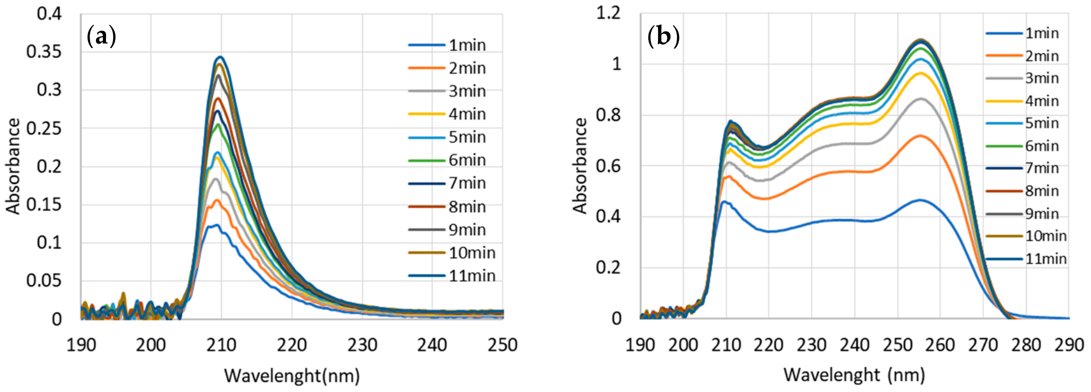

Figure S4. Absorption spectra in SBF of: (**a**) PPy_{90}/CA; (**b**) $PEDOT_{120}$/CA.

Table S3. Percentage of ibuprofen released into SBF according to the respective voltages applied after 11 minutes.

Systems	Voltage (V)/Diffusion	IBU (%)
PPy_{90} Rim System	-0.3	292
	+0.3	306
	+0.5	457
	+1.0	385
	Diffusion	298
PPy_{90} Single System	-1.5	298
	-0.3	311
	0	337
	+0.3	315
	+0.8	388
	+1.2	347
	Diffusion	300
$PEDOT_{120}$ Rim System	-0.3	372
	+0.3	443
	+0.5	505
	Diffusion	545
$PEDOT_{120}$ Single System	-0.3	488
	+0.3	665
	+0.8	724
	Diffusion	492
Mix Rim System	-0.3	378
	+0.3	342
	+0.8	459
	+1.2	475
	Diffusion	527

Table S4. Evaluation of linearity and working range of the UPLC-MS/MS method for IBU analysis.

Parameter	Linear interval	Working range
Number of calibration points (N)	7	6
Concentration range (µg/L)	0 – 14.5	1.5 – 14.5
Line equation	y = 16535.14x – 79.522	y = 16539.96x – 126.368
Coefficient of determination (R^2):	1.0000	0.9999
Coefficient of variation of the method (CVm, %)	0.51	0.57
Residue analysis (%)	[-3.7 – 0.0]	[-3.7 – 0.0]
Limit of detection (LOD), µg/L	0.11	0.12
Limit of quantification (LOQ), µg/L	0.36	0.40

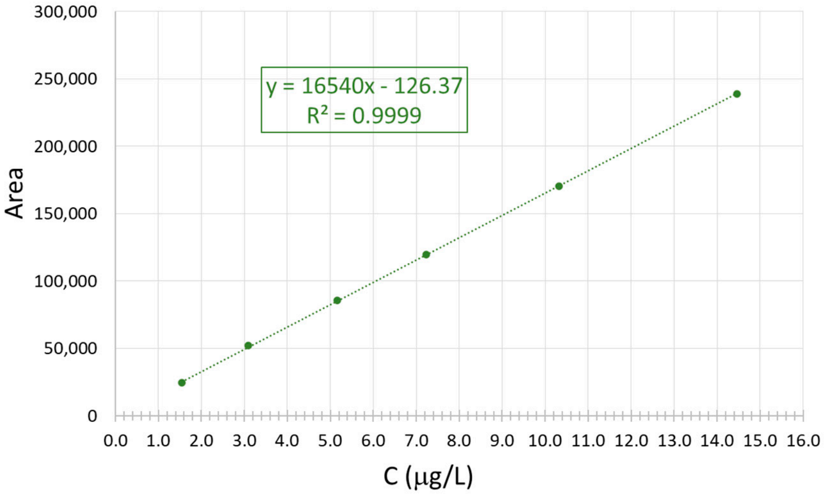

Figure S5. Working range of ibuprofen quantification by UPLC-MS/MS.

To minimize potential matrix effects, suppression or ion enrichment, samples were diluted 1:1 with ultrapure water. Consequently, the concentrations obtained by direct reading on the calibration curve, area versus concentration (µg/L), were multiplied by two.
